# Cardiovascular Collapse Requiring Veno-Arterial Extracorporeal Membrane Oxygenation and Lidocaine: A Case of Massive Bupropion Overdose

**DOI:** 10.7759/cureus.62873

**Published:** 2024-06-21

**Authors:** Nicole Reinsch, Ebaad Haq, Kartik Ramakrishna, Lindy Craft

**Affiliations:** 1 Department of Internal Medicine, Legacy Emanuel Medical Center, Portland, USA; 2 Department of Pulmonary Critical Care, Legacy Emanuel Medical Center, Portland, USA

**Keywords:** va-ecmo, lidocaine, cardiac arrest, qrs prolongation, cardiovascular collapse, impella cp, bupropion overdose

## Abstract

Bupropion is an antidepressant used in the treatment of major depressive disorder, seasonal affective disorder, nicotine addiction, and weight loss. It primarily functions via norepinephrine and dopamine reuptake inhibition. At toxic doses, bupropion can elicit seizures, as well as precipitate corrected QT interval (QTc) and QRS prolongation. We describe a case of an 18-year-old female who reportedly ingested 28 grams of extended-release bupropion, a dose much higher than in previously reported cases. Toxic ingestion precipitated status epilepticus, prolonged QTc, widened QRS, pulseless ventricular tachycardia (pVT), and subsequent cardiovascular collapse necessitating veno-arterial extracorporeal membrane oxygenation (ECMO) and Impella support. Historically, the cardiotoxic effects of bupropion toxicity have largely been treated with supportive care, sometimes requiring ECMO. This patient’s course was complicated by a widening QRS despite aggressive bicarbonate therapy and recurrent pVT, which was ultimately aborted with lidocaine. Neurological prognostication was further complicated by a lack of brainstem reflexes on the exam. With maximal supportive care, the patient was liberated from Impella, ECMO, and the ventilator by hospital day seven. At discharge, she was neurologically intact with full recovery of cardiac function. This case emphasizes the need for early consideration of transfer to an ECMO center in the setting of a bupropion overdose and offers a potentially effective treatment option for bupropion-induced ventricular arrhythmia.

## Introduction

Bupropion is an antidepressant that inhibits norepinephrine and dopamine reuptake. It is rapidly absorbed through the gastrointestinal tract with a half-life of up to 21 hours when chronically dosed and it is metabolized predominantly in the liver [1,2]. Encephalopathy, agitation, seizures, corrected QT interval (QTc) prolongation, as well as QRS widening, are notable toxic effects [1]. Here we discuss a case of intentional massive bupropion overdose (OD) resulting in brain death mimicry, recurrent arrhythmia, and cardiovascular collapse necessitating lidocaine, as well as mechanical circulatory support with veno-arterial (VA) extracorporeal membrane oxygenation (ECMO) and Impella.

## Case presentation

An 18-year-old female with a history of anxiety, depression, and insomnia presented to her local emergency room (ER) from urgent care two hours after an intentional OD of bupropion extended-release. The patient reported taking 28 grams (g); a review of pharmacy records demonstrated recurrent monthly fills of 13.5 g, though it was unclear if the patient had been adherent before this event. On initial evaluation, she was non-toxic appearing with stable vital signs and a QTc of 478, as well as a QRS of 84. Her urine drug screen was positive for marijuana. She was admitted for serial electrocardiograms (EKG) per the recommendation of poison control.

Three hours after arrival to the ER she had three witnessed tonic-clonic seizures followed by apnea requiring intubation. A midazolam infusion was started and the patient was loaded with levetiracetam, as well as fosphenytoin. During central line placement, the patient developed brief pulseless electrical activity (PEA) arrest, with the return of spontaneous circulation (ROSC) following two rounds of compressions and a dose of epinephrine. Given the potential for further decline, transfer to an ECMO center was recommended by poison control.

On hospital day (HD) two the patient was transferred to our facility and arrived on low-dose norepinephrine. The initial exam was remarkable for multiple absent brainstem reflexes; pupils were dilated and non-reactive, and there was no cough, gag, or oculocephalic reflex. Electroencephalograms were negative for epileptic activity. The QTc and QRS were 523 and 116, respectively, and the patient was started on continuous sodium bicarbonate. An echocardiogram on arrival was remarkable for an ejection fraction (EF) of 60-65% with normal right ventricular (RV) systolic function.

**Figure 1:**
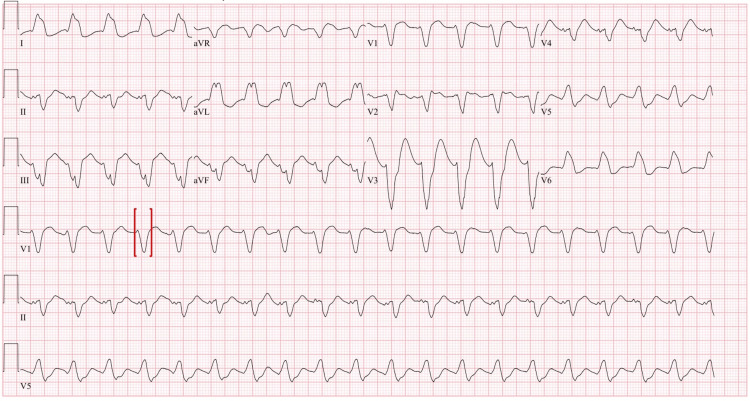
EKG prior to arrest on ECMO Red brackets: prolonged QRS of 164 aVR: augmented vector right; aVL: augmented vector left; aVF: augmented vector foot; ECMO: extracorporeal membrane oxygenation

Several hours after arrival the patient developed pulseless ventricular tachycardia (pVT), with ROSC following one round of compressions and defibrillation. Post-arrest she required escalating doses of vasopressor support and was placed on VA ECMO for cardiovascular collapse. Intravenous lipid emulsion therapy was considered though not pursued due to the initiation of ECMO. On HD three, she developed pVT while on ECMO; the QTc before the arrest was 650 with a QRS of 164 and a morphology that resembled a left bundle branch block (Figure 1).

**Figure 2:**
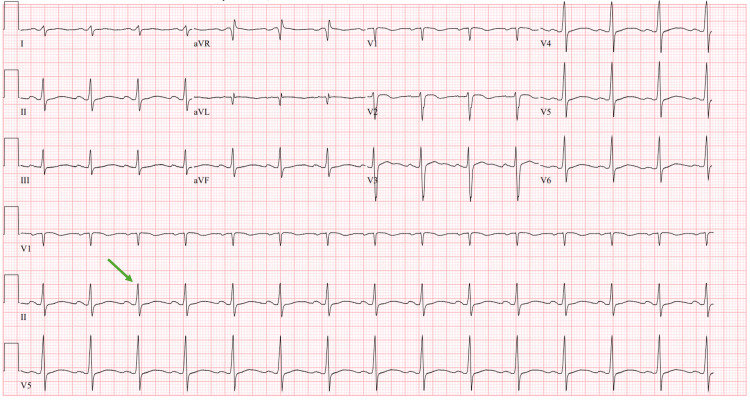
EKG following the administration of lidocaine Green arrow:* *narrowed QRS of 104 aVR: augmented vector right; aVL: augmented vector left; aVF: augmented vector foot

During the arrest on ECMO, a lidocaine bolus and infusion were started and sinus rhythm was regained without any shocks delivered. Following lidocaine administration, the QTc and QRS had narrowed to 534 and 104, respectively (Figure 2). She had no recurrence of ventricular tachycardia (VT) while on the lidocaine infusion. A repeat echocardiogram was now remarkable for severe global hypokinesis of the left ventricle (LV) with an EF of 10% and mildly reduced RV function. Following rhythm stabilization, Impella CP was placed for additional LV support. Remarkably, the patient’s renal and hepatic function remained intact.

On HD four, the patient was blinking to threat. Sedation, vasopressors, and mechanical support were starting to be weaned. On HD five, pupils were reactive and by HD six the patient was spontaneously opening her eyes, tracking, and following commands. The patient was successfully decannulated from ECMO and extubated on HD seven. The Impella was removed the following day. After discontinuation of cardiovascular support, a repeat echocardiogram revealed recovery of baseline cardiac function (Video 1). Antiarrhythmics and antiepileptic drugs were eventually stopped. She was discharged on HD17, neurologically intact, with plans for intensive outpatient psychiatric therapy.

**Video 1:** Recovery of baseline cardiac function prior to discharge

## Discussion

This case highlights the significant toxic effects of a massive bupropion OD of reportedly 28 g. There have been multiple case reports documenting the association of bupropion with brain death mimicry [3], which we too observed in this patient. Perhaps most notable about this case was the severity of cardiovascular toxicity. There is no antidote for a bupropion OD and patients are largely managed with supportive measures; activated charcoal and whole bowel irrigation can be considered in some [4]. Sinus tachycardia and QRS widening are the most common cardiac manifestations of bupropion toxicity [5] and are generally well tolerated with supportive care while the medication metabolizes.

This patient demonstrated QTc and QRS prolongation, multiple cardiac arrests, as well as biventricular dysfunction complicated by cardiogenic shock. The patient's hemodynamic instability and EKG changes persisted despite electrolyte replacement, sodium bicarbonate, and escalating vasopressor support. Intravenous lipid emulsion therapy can be utilized in those with hemodynamic instability and may have had some effectiveness in other cases of bupropion OD [6,7]; this was considered for our patient but was not pursued given the immediate availability of ECMO and the potential for lipid emulsion therapy to complicate the use of ECMO [8].

The patient was cannulated for VA ECMO given the severity of their cardiovascular collapse. ECMO has been successfully utilized in patients with cardiovascular decompensation due to acute drug intoxication secondary to antiarrhythmics, nodal-blocking agents, and antidepressants [9]. A retrospective cohort analysis in France revealed improved survival in poisoned patients who had experienced cardiac arrest and shock and who received extracorporeal life support (86%) versus those who received conventional therapies (48%) [10]. Given the potentially lengthy half-life of bupropion, a case can be made for early ECMO consideration, including transport to a capable facility, in those demonstrating QRS widening, hypotension, shock, or dysrhythmia. Previously described cases of refractory shock necessitating VA ECMO in the setting of a bupropion OD have demonstrated that the toxic effects are likely to resolve if the patient can be sufficiently supported [11].

While on ECMO, our patient developed sustained VT with loss of pulsatility for the second time likely due to the QTc and QRS prolonging effects of bupropion, neither of which were meaningfully affected by sodium bicarbonate. A small retrospective study demonstrated that sodium bicarbonate did not significantly decrease QRS widening in the setting of a bupropion OD [12]. While QRS widening secondary to a sodium channel blocker can respond favorably to sodium bicarbonate, Caillier et al. demonstrated that bupropion does not significantly affect the cardiac sodium channel, rather it prolongs the QRS via altering gap junction cardiac communication [5]. O’Brien et al. report the case of a patient with bupropion OD who required 13 defibrillations for recurrent VT in the first 10 hours on VA ECMO [13]. In the case of our patient, pVT was aborted rapidly after lidocaine, a cardiac sodium channel inhibitor, and did not return. There is one other case report of a patient with a bupropion OD developing recurrent VT followed by ventricular fibrillation, successfully treated with lidocaine [14]. While reports on the use of lidocaine in bupropion-induced ventricular arrhythmia are limited, there may be a role in the setting of cardiotoxicity.

## Conclusions

Cardiovascular collapse is a potential complication of bupropion OD. While data is limited, this case report, amongst others, supports the use of VA ECMO in patients demonstrating evidence of cardiotoxicity and stresses the importance of early consideration of such interventions. Similarly, though anecdotally, this case suggests there may be a benefit to the use of lidocaine for ventricular dysrhythmias secondary to bupropion toxicity. Given the potential for brain death mimicry, it is imperative to allow sufficient time for bupropion metabolism before neurological prognostication, especially in the setting of cardiac arrest.
